# Effects of Dietary Rumen-Undegradable Protein and Protein Levels on Growth Performance, Fermentation Parameters, Slaughter Performance, and Meat Quality on Fattening Hu Sheep

**DOI:** 10.3390/ani16152407

**Published:** 2026-08-04

**Authors:** Changxin Tian, Zhibo Wang, Shengdi Hu, Biao Yun, Zhaojin Liu, Xueqiao Qian, Zhixiong He

**Affiliations:** 1 Animal Husbandry and Fisheries Research Center of Guangdong Haid Group Co., Ltd., Guangzhou 511400, China; tianzx2@haid.com.cn (C.T.);; 2 National Engineering Laboratory for Pollution Control and Waste Utilization in Livestock and Poultry Production, Hunan Provincial Key Laboratory of Animal Nutrition & Physiology and Metabolism, Scientific Observing and Experimental Station of Animal Nutrition and Feed Science in South-Central, Ministry of Agriculture, Institute of Subtropical Agriculture, The Chinese Academy of Sciences, Changsha 410125, China

**Keywords:** fattening sheep, growth performance, meat quality, rumen-undegradable protein, rumen fermentation

## Abstract

Optimizing protein nutrition in ruminants is vital for enhancing growth performance, meat quality, and overall production efficiency. However, the precise protein requirement for ruminants remains unclear. Two experimental trials were conducted to investigate the impact of varying levels of rumen-undegradable protein (RUP) on in situ feed degradability, growth performance, meat quality, and rumen fermentation parameters in fattening Hu sheep. In EXP 1, six Hu sheep were fitted with rumen fistulas to measure rumen degradation characteristics of diets varying levels of crude protein (CP) (13%, 14%, 15%, and 16% X RUP: CP 40%, 45%, 50%). The results showed that the ruminal DM and GE degradation had no difference between the experimental diets (*p* > 0.05). However, the CP degradation rate gradually decreased as the dietary RUP proportion increased (*p* < 0.05). In EXP 2, a double factorial experiment was conducted using 720 male Hu sheep, divided into twelve dietary treatments with varying levels of crude protein (CP) (13%, 14%, 15%, and 16%) and RUP (40%, 45%, and 50%). Key performance indicators such as body weight gain (BWG), average daily gain (ADG), average daily feed intake (ADFI), and feed conversion ratio (FCR) were measured at the beginning and end of the trial. Post-slaughter, meat quality attributes, including drip loss and initial pH of the longissimus dorsi muscle, were assessed after 40 days of the trial. Rumen fluid samples were analyzed for volatile fatty acid (VFA) profiles to evaluate rumen fermentation efficiency. The results demonstrated that increasing RUP levels significantly improved BWG and ADG (*p* < 0.05) without affecting ADFI and FCR. Although dietary protein levels had minimal impact, higher RUP levels were associated with a trend toward increased final body weight (*p* = 0.09). Dietary protein levels and the RUP: CP ratio had no significant effect on meat quality (*p* > 0.05). Ruminal acetate, valerate, and iso-valerate concentrations were reduced as RUP: CP increased (*p* < 0.05), and propionate concentration showed a similar trend (*p* = 0.062). In conclusion, optimizing RUP levels in the diets of fattening Hu sheep significantly enhances growth performance without compromising feed efficiency or the overall rumen fermentation process. These findings provide a foundation for developing feeding strategies that maximize production efficiency in sheep farming.

## Simple Summary

Protein nutrition is essential for efficient sheep production, but the optimal protein requirement remains unclear. This study examined whether adjusting the proportion of protein that escapes rumen breakdown could improve growth and meat quality without compromising digestive health in fattening Hu sheep. We conducted a rumen degradation trial using fistulated sheep followed by a feeding trial, with 720 sheep receiving diets with different protein levels and rumen-undegradable protein ratios. Increasing rumen-undegradable protein significantly improved body weight gain and average daily gain, without affecting feed intake, feed efficiency, or meat quality. Rumen fermentation remained stable. These findings suggest that optimizing dietary protein fractions can enhance growth performance while maintaining digestive function, offering practical strategies for more efficient and sustainable sheep farming. Further validation under commercial conditions is recommended.

## 1. Introduction

Consumption of high-quality dietary protein is essential for improving sheep nutrition, growth, development, and health. Ruminal microorganisms degrade ingested proteins into peptides, amino acids, and ammonia, which are then used to synthesize microbial crude proteins (MCPs), providing a new protein source for ruminant growth [1]. In ruminants, maintaining the balance between the rumen-degradable protein (RDP) and rumen-undegradable protein (RUP) is crucial as it influences microbial activity and the availability of essential amino acids for absorption in the small intestine. Therefore, a well-balanced protein diet is pivotal for enhancing growth performance and maintaining robust health in fattening sheep.

Studies on fattening sheep indicate that moderate increases in dietary protein can enhance growth performance by improving growth rates, feed conversion efficiency, muscle protein synthesis, and amino acid availability. However, most dietary proteins ingested by ruminants are extensively degraded in the rumen. Excessively high-protein diets may lead to nitrogen wastage as rumen microorganisms efficiently degrade proteins to produce ammonia nitrogen, potentially causing inefficiencies in protein utilization [2]. Therefore, ruminant protein requirements are categorized into RDP and RUP. RDP is essential for supporting the growth and activity of rumen microbes that biotransform fibrous feed into microbial protein, volatile fatty acids, and other nutrients [3]. In contrast, RUP escapes rumen degradation and is digested in the small intestine, supplying essential amino acids directly for absorption and utilization by the ruminant [4,5]. Studies have shown that adequate RUP in the diet of ruminants is associated with improved growth performance, milk production, and reproductive performance [6,7,8,9], as RUP provides higher amino acid (AA) content that enters the small intestine. In addition, the amino acids available through RUP can be even more easily digested than proteins from microbial sources in the small intestine of sheep [10]. In fast-growing and high-yielding animals, microbial protein supply is limited. Therefore, to meet the demand, it is necessary to provide protein in the form of RDP. Hence, the optimal ratio of RDP to RUP ensures that sufficient nitrogen is available for microbial synthesis while providing adequate absorbable amino acids for animal growth and productivity.

Currently, the optimal dietary protein content and RUP ratios in ruminant nutrition, particularly for fattening sheep, have limitations. Many studies focus on generic CP levels without differentiating the specific contributions of RDP and RUP from growth performance and health outcomes. Moreover, there is a lack of comprehensive studies that explore the optimal RUP-to-CP ratio under varying physiological states and production systems, particularly for fattening sheep. This study aims to address these gaps by investigating the interrelationship between dietary protein content and the RUP ratio specific to fattening sheep. By providing a detailed analysis of how these dietary factors influence growth performance, rumen fermentation parameters, and overall metabolic health, this study aims to enhance the understanding of protein utilization efficiency, optimize animal performance, and reduce environmental impact through better nitrogen management.

## 2. Materials and Methods

### 2.1. Animal Ethics Statement

All animal care and procedures were approved by the Animal Care and Use Committee of the Institute of Subtropical Agriculture in compliance with the Regulations for the Administration of Affairs Concerning Experimental Animals on 16 January 2019 (No. ISA-2019-0116). The experiment was conducted at the Institute of Subtropical Agriculture, Chinese Academy of Sciences, Changsha, China.

### 2.2. Experimental Design and Diets

A double-factorial experiment was designed to study the effects of dietary protein levels (CP: 13%, 14%, 15%, and 16%) and RUP proportion (RUP: CP: 40%, 45%, and 50%) on the growth performance and gut fermentation parameters of fattening sheep. Table 1 presents the ingredient composition of the experimental diets. According to the guidelines set by the NRC (2001) [11], all experimental diets were designed to meet or exceed the nutritional requirements for sheep at a body weight of 30 kg.

### 2.3. In Situ Degradability of Diets (EXP1)

Six Hu sheep (six months, average body weight: 30.7 ± 1.2 kg), fitted with rumen fistulas to measure rumen degradation characteristics, were housed in the laboratory animal room at the Institute of Subtropical Agriculture, Chinese Academy of Sciences (China). These sheep were fed a diet with a 67:33 concentrate-to-roughage ratio at 08:00 and 16:00, with water provided ad libitum. Nylon bags (ANKOM Technology, Macedon, NY, USA) used for the experiment measured 10 cm × 6 cm with an aperture size of 48 µm (300 mesh). After being placed in the rumen, the nylon bags were retrieved, repeatedly washed, dried in an oven at 65 °C for two days, equilibrated for one day, and then weighed. Each diet using exp2 (*n* = 12 diets), carefully weighed to three grams, was placed in a separate nylon bag. These bags were attached to a flexible semi-plastic hose and inserted into the rumens of six different sheep, with duplicate samples placed in each rumen at each time point (0 and 48 h; total time points = 2). The 12 ingredients were divided into 2 runs, with 6 ingredients per run. After incubation, the nylon bags were removed from the rumen and rinsed until the rinse water was clear, ensuring the elimination of residual ruminal contents and bacteria to halt microbial activity. The bags and their residues were then dried at 65 °C until they reached a constant weight. For the 0 h incubation samples, only washing was performed under the same conditions. The residues were weighed and ground through a 1 mm sieve, and then mixed and analyzed for nutrient content including dry matter (DM), crude protein (CP), and gross energy (GE). DM was determined by drying at 105 °C to a constant weight. CP was calculated as N × 6.25, following nitrogen analysis by Kjeldahl digestion and distillation [12]. Gross energy (GE) was determined using an isothermal automatic calorimeter (5E-AC8018; Changsha Kaiyuan Instruments Co., Changsha, China). The chemical composition of the experimental diets, including dry matter (DM), crude protein (CP), ether extract (EE), and ash, was determined according to standard methods [13]. Neutral detergent fiber (NDF) and acid detergent fiber (ADF) were analyzed following the previous procedures [14].

### 2.4. Experimental Animal Trial (EXP2)

Seven hundred and twenty sheep (Hu sheep, local breed) with similar body weights (30.7 ± 1.2 kg) and that were 0.6 years old were selected and randomly assigned into twelve dietary treatments. For growth performance, data were measured at the pen level (10 lambs per pen; 6 pens per treatment) and analyzed by the mean of each pen. All animals were penned in groups and fed with a 67:33 ratio of concentrate to forage twice per day (08:00 and 16:00), with free access to water. The groundnut straw and husks were used as the forage source (Table 1). The experimental period lasted for 40 days. The feed intake per pen was recorded every day, and the ADFI and FCR were calculated for each pen at the end of the trial (on d41). Seventy-two sheep (six sheep per treatment, 12 treatments, randomly selected) were slaughtered at the end of the trial (on d41). The selected animals were fasted for 12 h, and then slaughtered, exsanguinated, eviscerated, and split. The body weight before slaughter and carcass weight were recorded (*n* = 72 sheep). After slaughter, the eye muscle area was outlined on a grid paper between the 12th and 13th rib cuts, and the area was then calculated (*n* = 72 sheep). The contents of the rumen and ileum were collected, flash-frozen in liquid nitrogen, and stored at −80 °C (*n* = 72 sheep). The longissimus dorsi muscles of the left carcass were vacuum-packed and chilled for 24 h (2–4 °C) for later determination of meat quality characteristics (*n* = 72 sheep).

### 2.5. Meat Quality Characteristics

Muscle pH was recorded using a pH meter (STARTER2100, Shanghai, China). Color coordinates (lightness, L*; redness, a*; yellowness, b*) were determined at three random locations using a colorimeter (CR-410, Konica Minolta, Tokyo, Japan). The sheer force of the muscle was measured using a muscle tenderness meter (TA500 Lloyd Texture Analyzer, fitted with a triangular Warner–Bratzler shear (Lloyd instruments, Bognor Regis, UK). Drip loss was calculated as the difference in weight before and after storage, divided by the initial weight, as previously described [11].

### 2.6. Volatile Fatty Acids Analysis

After thawing the rumen fluid, 0.5 g of the sample was accurately weighed in a 2 mL centrifuge tube. Then, 1.5 mL of distilled water was added and mixed for 30 min before being centrifuged at 15,000 r/min for 15 min. Next, 2 mL of rumen fluid was centrifuged at 15,000× *g* for 10 min at 4 °C. Then, 1 mL of supernatant was taken, and 0.2 mL of 25% metaphosphoric acid was added, mixed well, and left to incubate at 4 °C for 30 min. It was then centrifuged at 15,000 r/min for 15 min, and the supernatant was filtered through a 0.22 μm filter membrane. After thawing the ileal digesta, approximately 0.5 g of the sample was accurately weighed in a 2 mL centrifuge tube. Then, 1.5 mL of distilled water was added, and the mixture was vortexed for 5 min to homogenize the digesta, followed by mixing for 30 min before centrifugation at 15,000 r/min for 15 min. Subsequently, 2 mL of the supernatant was carefully transferred to a new centrifuge tube and centrifuged at 15,000× *g* for 10 min at 4 °C. Then, 1 mL of supernatant was taken, and 0.2 mL of 25% metaphosphoric acid was added, mixed well, and left to incubate at 4 °C for 30 min. It was then centrifuged at 15,000 r/min for 15 min, and the supernatant was filtered through a 0.22 μm filter membrane. The contents of acetic acid, propionic acid, iso-butyric acid, butyric acid, isovaleric acid, and valeric acid were determined by gas chromatography–mass spectrometry. Both the dilution factor for rumen fluid and ileal digesta were 1.2-fold.

### 2.7. Statistical Analysis

For Exp. 1 (in situ rumen degradability), statistical analyses were performed using a two-way repeated-measures ANOVA (CP × RUP:CP) with SPSS 26.0 (IBM Corp., Armonk, NY, USA). The animals were treated as a random effect to account for repeated measurements taken from the same individual over time.

For Exp. 2 (growth performance, slaughter performance, and meat quality), statistical analyses were performed using a two-way ANOVA (CP × RUP: CP) with SPSS 26.0, where CP and RUP:CP were included as fixed effects, along with their interaction (CP × RUP:CP). For feed intake and FCR, pen served as the experimental unit (*n* = 72, six pens per treatment for 12 treatments). For slaughter performance and meat quality, individual animals served as the experimental unit (*n* = 72, six sheep per treatment for 12 treatments).

When significant main effects or interactions were detected, means were separated using Duncan’s multiple range test. Statistical significance was declared at *p* < 0.05, and a tendency was considered at 0.05 ≤ *p* ≤ 0.10. All data are presented as mean ± standard deviation (SD).

## 3. Results

### 3.1. In Situ Rumen Degradability of Different Diets (EXP1)

Table 2 shows the rumen degradability of dry matter (DM), crude protein (CP), and gross energy (GE) of the experimental diets. The ruminal DM and GE degradation had no difference between the experimental diets (*p* > 0.05). However, the CP degradation rate gradually decreased as the dietary RUP proportion increased (*p* < 0.05).

### 3.2. Growth and Slaughter Performance (EXP2)

As shown in Table 3, the two-way ANOVA revealed no significant CP × RUP:CP interactions for any of the parameters measured (*p* > 0.05 for all), indicating that the effects of dietary CP level and RUP: CP ratio were independent of each other. Dietary protein level did not affect the BW gain, ADFI, ADG, and FCR in fattening sheep (*p* > 0.05). As the proportion of RUP in the diet increased from 40% to 50%, the BW gain and ADG increased significantly (*p* < 0.05) and the final BW tended to increase (*p* = 0.09). Dietary RUP level had no effect on ADFI and FCR. No significant differences in carcass weight, dressing percentage, or eye muscle area among the different dietary treatment groups were found (*p* > 0.05). However, the dressing percentage tended to increase with a higher proportion of RUP in the diet (*p* = 0.086).

### 3.3. Meat Quality

As shown in Table 4, dietary protein levels and RUP: CP ratio had no significant effect on drip loss, shear force, meat color (L*, a*, b*), and pH of the longissimus dorsi muscle in Hu sheep (*p* > 0.05).

### 3.4. The Content of Volatile Fatty Acids in the Rumen and Ileum

Data on the volatile fatty acids in the rumen and ileum are presented in Table 5 and Table 6. The two-way ANOVA revealed no significant CP × RUP:CP interactions for any of the parameters measured (*p* > 0.05 for all), indicating that the effects of dietary CP level and RUP: CP ratio were independent of each other. In the rumen, there were no significant differences in total volatile fatty acids (TVFA) among the dietary treatment groups (*p* > 0.05). The concentrations of acetate, propionate, iso-butyrate, butyrate, and valerate were not affected by the dietary protein levels (*p* > 0.05). However, as the proportion of RUP in the diet increased, the ruminal concentrations of acetate, valerate, and iso-valerate significantly decreased (*p* < 0.05), and the concentration of propionate showed a similar trend (*p* = 0.062). In the ileum, dietary protein levels and the proportion of RUP had no significant effect on the concentrations of TVFA (*p* > 0.05).

## 4. Discussion

Rumen-undegradable protein (RUP) plays a crucial role in providing essential amino acids to ruminants by bypassing ruminal fermentation and being directly digested in the intestines, thereby significantly influencing their performance [15]. In this study, we formulated diets with varying protein levels and RUP proportions. The in situ rumen degradability test indicated no significant differences in the degradation rates of DM and GE among the experimental diets, suggesting that variations in dietary RUP do not significantly affect the ruminal degradability of these components. Similarly, Pina, Valadares [16], found that supplementing with RUP at either 25% or 40% of CP had no significant effect on the digestibility of DM, OM, neutral detergent fiber crude protein (NDFcp), and total digestible nutrients (TDN) in Nellore heifers. These consistent results might be attributed to the inherent stability and efficiency of the ruminal microbial ecosystem in breaking down these components uniformly, regardless of RUP levels. However, the degradation rate of crude protein (CP) significantly decreased with the increasing dietary levels of RUP. This expected reduction indicates that higher RUP proportions might result in a greater fraction of dietary protein escaping ruminal degradation, which may be conducive to improving the availability of amino acids for post-ruminal digestion.

In the present study, although metabolizable energy (ME) and microbial protein production (MCP) were not directly measured, the FME levels across treatments were estimated to be largely comparable based on measured gross energy (GE) values and published energy conversion coefficients for sheep (NRC, 2001) [11]. Therefore, it can be reasonably inferred that when CP level was increased from 13% to 16%, the additional RDP could not be effectively utilized by rumen microbes due to limited FME supply, resulting in no further improvement in growth performance. This inference is consistent with our finding that CP level had no significant effect on ADG or BWG.

Dietary protein level is an important factor influencing body tissue growth. In this study, intake levels were consistent across experimental groups, likely due to the similar energy content of the diet. Allen, Bradford [17], noted that energy is the primary limiting factor for intake in ruminants. The average NDF content in the basal diet (33.89%) may have also contributed to the stable dry matter intake across treatments. Consequently, increasing dietary RUP content did not impact intake. However, despite comparable intake levels, average daily gain (ADG) and body weight gain (BWG) improved with a higher RUP:CP ratio in fattening sheep. This finding is consistent with previous studies [7], which indicate that a higher dietary RUP content enhances ADG in growing lambs. However, increasing dietary protein levels did not have a similar effect. The finding is consistent with studies conducted by Kaya, Ünal [18], and Wang, Xu [19], in which changes in protein levels (CP ≥ 12%) did not typically result in significant differences in growth parameters in sheep. This lack of effect indicates that the provided protein levels were within a specific optimal range required for maintaining the growth performance of fattening sheep, and further increases did not provide additional benefits. The beneficial effect of RUP implied that higher RUP levels contribute positively to growth performance, likely driven by an improved quality of post-ruminally available protein that more effectively meets metabolic demands. Furthermore, dietary energy content and protein levels are key factors influencing carcass traits in meat-producing animals [20,21]. In this experiment, energy content and intake levels were consistent across treatments, while dietary CP levels and RUP: CP ratios varied, which may result in no significant differences among groups in slaughter parameters and meat quality traits in the present study. Francisco, Janíček [22], showed that the diet compositions were the main factors associated with carcass traits. In this study, dietary protein levels and RUP: CP ratios did not significantly influence drip loss, shear force, meat color (L*, a*, b*), and the pH of muscle. These results are consistent with previous findings [23,24], which indicated uniform meat quality characteristics across a defined range of dietary protein levels, regardless of RUP levels.

Volatile fatty acids (VFAs) are key fermentation by-products and serve as a primary energy source for ruminants. The present study revealed no significant differences in total VFAs among the dietary treatment groups in the rumen, regardless of protein levels. Similarly, the proportions of acetate, propionate, iso-butyrate, butyrate, and valerate were not significantly affected by protein levels, highlighting a stable fermentation pattern. These results are consistent with the observations of Jo, Kim [25], who also reported stable VFA profiles across varying protein diets in ruminants. However, a decreasing RUP: CP ratio resulted in higher concentrations of ruminal acetate, valerate, and iso-valerate, while the acetate/propionate ratio increased. Soliva, Amelchanka [26], indicated that the requirement for RDP to achieve optimal ruminal fermentation of organic matter and fiber may differ when feeding with various fibrous feeds. Similarly, feeding a high RUP:CP-ratio diet to growing lambs decreased propionate concentration in the rumen. These alterations underscore microbial adaptations to dietary nitrogen balance, which favor an efficient fermentation environment conducive to better feed utilization.

## 5. Conclusions

This study elucidates the pivotal role of dietary RUP content in enhancing growth performance in fattening Hu sheep. Although dietary protein levels had minimal impact on measured parameters per se, strategic adjustments in RUP content result in significant advantages. The nuanced shifts in VFA profiles with higher RUP levels underscore microbial fermentation adaptations, further promoting a more efficient digestive framework. Further research is recommended to explore the long-term implications and the broader economic viability of these dietary adjustments in commercial sheep production.

## Figures and Tables

**Table 1:** Ingredients composition and nutrients level of the experimental diets.

Component	CP: 13%			CP: 14%			CP: 15%			CP: 16%		
	RUP: 50%	RUP: 45%	RUP: 40%	RUP: 50%	RUP: 45%	RUP: 40%	RUP: 50%	RUP: 45%	RUP: 40%	RUP: 50%	RUP: 45%	RUP: 40%
Ingredients composition, %												
Maize	33	33	33	33	33	33	33	33	33	33	33	33
Corn germ meal	18.5	18.5	18.5	15.5	19	19	15	16.9	16.3	17.5	22	24.2
Corn gluten meal	3.8	1.5	-	4.5	0.7	-	7	1	-	5.5	2.6	-
Corn gluten feed	6	10	10.9	8	8.3	8.3	8	12	12	5	5	5
Groundnut husks	18	18	18	18	18	18	18	18	18	18	18	18
Groundnut straw	15	15	15	15	15	15	15	15	15	15	15	15
NH_4_Cl	0.2	0.2	0.2	0.2	0.2	0.2	0.2	0.2	0.2	0.2	0.2	0.2
NaCl	0.4	0.4	0.4	0.4	0.4	0.4	0.4	0.4	0.4	0.4	0.4	0.4
NaHCO_3_	1	1	1	1	1	1	1	1	1	1	1	1
CaCO_3_	1.8	1.8	1.8	1.8	1.8	1.8	1.8	1.8	1.8	1.8	1.8	1.8
Premix ^1^	0.6	0.6	0.6	0.6	0.6	0.6	0.6	0.6	0.6	0.6	0.6	0.6
Bentonite	1.7	-	-	2	2	2	-	-	1	2	-	-
Urea	-	-	0.6	-	-	0.7	-	0.1	0.7	-	0.4	0.8
Total	100	100	100	100	100	100	100	100	100	100	100	100
Nutrient levels ^2^, DM basis %												
CP	13.1	13	13.1	14.1	13.9	14.2	15.1	15	15.1	16	16	16
EE	4.4	4.5	4.5	4.5	4.5	4.6	4.8	5.0	5.0	5.0	5.0	5.1
NDF	35.3	35.3	35.5	33.4	33.1	33.9	33.5	33.5	33.3	32.9	33.2	33.4
ADF	22.4	22.3	22.3	20.6	20	20	20	20.1	20.6	19.9	20.2	20.5
RUP:CP ^3^, %	50	45	40	50	45	40	50	45	40	50	45	40
GE ^4^, MJ/kg	16.6	16.6	16.5	16.1	16.1	16.1	16.7	16.7	16.7	16.8	16.8	16.8

^1^ Premix composition per kg diet: 68 mg FeSO_4_ H_2_O, 44 mg CuSO_4_ 5H_2_O, 411 μg CoC1_2_ 6H_2_O, 1.70 mg KIO_3_, 211 mg MnSO_4_ H_2_O, 126 mg ZnSO_4_ H_2_O, 56 µg Na_2_SeO_3_, 462 mg MgSO_4_ 7H_2_O, 737 IU vitamin A, 8.29 mg vitamin E, 4.0 g NaHCO_3_, and 5.1 g carrier zeolite powder. ^2^ Nutrient levels were measured values. ^3^ RUP: CP (%): The value was calculated by NRC 2001 [11]. ^4^ GE: Gross energy.

**Table 2:** The 48 h rumen degradability of the experimental diets.

Factorial Experiment		Variables		
Protein Level (% DM)	RUP Level (% CP)	DMD, %	CPD, %	GED, %
13	50	55.4 ± 12.1	41.1 ± 10.3	71.2 ± 1.20
	45	63.2 ± 8.70	43.1 ± 11.2	70.3 ± 2.34
	40	63.6 ± 12.2	52.1 ± 8.42	70.1 ± 6.17
14	50	59.2 ± 10.1	52.1 ± 8.40	67.5 ± 2.18
	45	60.6 ± 10.0	39.9 ± 10.3	74.4 ± 8.24
	40	69.6 ± 2.91	47.9 ± 12.2	79.2 ± 2.30
15	50	64.8 ± 11.1	40.1 ± 12.2	73.2 ± 10.3
	45	64.7 ± 14.1	44.3 ± 10.3	72.4 ± 10.2
	40	44.4 ± 15.1	50.1 ± 10.2	61.6 ± 10.3
16	50	58.9 ± 14.2	45.3 ± 10.3	63.2 ± 4.12
	45	69.8 ± 2.28	46.1 ± 1.27	74.2 ± 1.63
	40	52.3 ± 11.3	52.3 ± 2.38	67.2 ± 14.2
*p* value				
CP		0.272	0.251	0.440
RUP		0.140	0.017	0.426
CP*RUP		0.565	0.125	0.561

All data are presented as mean ± standard deviation (SD), *n* = 144.

**Table 3:** Effect of dietary RUP ratio on growth performance and slaughter performance in fattening HU sheep.

Factorial Experiment		Variables								
Protein Level (% DM)	RUP Level (% CP)	IBW (kg)	FBW (kg)	BWG (kg)	ADFI (kg/d)	ADG (g/d)	FCR	CW (kg)	DP (%)	EMA (cm^2^)
13	50	32.2 ± 4.15	43.5 ± 2.71	11.3 ± 1.33	2.11 ± 0.46	283 ± 28.4	7.47 ± 2.11	19.5 ± 1.30	50.3 ± 1.32	17.1 ± 1.14
	45	30.7 ± 3.81	41.2 ± 4.43	10.3 ± 0.92	2.09 ± 0.28	257 ± 26.7	7.66 ± 1.45	18.8 ± 2.11	49.8 ± 0.86	16.3 ± 0.76
	40	31.5 ± 3.86	40.3 ± 4.63	10.8 ± 1.73	2.08 ± 0.16	270 ± 55.2	7.73 ± 2.43	18.7 ± 2.31	48.9 ± 2.13	16.9 ± 0.82
14	50	30.1 ± 3.70	41.7 ± 4.91	11.6 ± 1.85	2.10 ± 0.17	285 ± 47.6	7.10 ± 2.88	20.4 ± 2.45	51.6 ± 1.67	18.2 ± 0.46
	45	31.1 ± 3.80	41.6 ± 4.94	10.5 ± 1.14	1.98 ± 0.19	261 ± 28.4	7.65 ± 1.15	19.9 ± 2.55	49.5 ± 0.86	17.9 ± 1.03
	40	31.2 ± 3.40	41.6 ± 4.21	10.4 ± 0.82	2.06 ± 0.23	258 ± 17.2	7.62 ± 1.21	19.5 ± 2.02	49.1 ± 2.13	17.9 ± 0.58
15	50	31.1 ± 3.92	42.4 ± 3.57	11.3 ± 1.10	2.05 ± 0.24	279 ± 20.1	7.35 ± 1.80	21.3 ± 1.72	52.0 ± 2.45	18.5 ± 0.89
	45	32.3 ± 3.57	43.8 ± 4.25	11.5 ± 1.32	2.10 ± 0.15	285 ± 27.6	7.44 ± 2.01	21.0 ± 2.06	50.2 ± 2.68	18.7 ± 0.75
	40	31.5 ± 4.16	42.5 ± 4.20	11.0 ± 1.43	2.12 ± 0.14	263 ± 17.7	7.20 ± 1.22	20.4 ± 2.14	49.4 ± 1.23	17.9 ± 0.47
16	50	30.9 ± 3.62	41.2 ± 4.10	10.8 ± 1.33	1.97 ± 0.16	275 ± 8.42	7.12 ± 1.43	21.4 ± 1.88	52.2 ± 1.00	19.1 ± 0.86
	45	30.8 ± 3.65	41.6 ± 4.85	10.8 ± 0.82	1.96 ± 0.14	269 ± 28.9	7.31 ± 1.27	21.5 ± 2.52	51.4 ± 1.84	18.8 ± 0.57
	40	31.1 ± 3.71	41.4 ± 4.13	10.3 ± 0.61	1.99 ± 0.16	257 ± 44.2	7.42 ± 1.01	21.1 ± 2.17	50.2 ± 2.23	18.3 ± 0.49
*p* value										
CP		0.972	0.384	0.841	0.165	0.827	0.448	0.090	0.080	0.256
RUP		0.427	0.090	0.042	0.981	0.044	0.539	0.095	0.086	0.437
CP*RUP		0.996	0.952	0.525	0.681	0.535	0.873	0.743	0.134	0.581

*n* = 72 (12 treatments; six pens per treatment); IBW: initial body weight; FBW: final body weight; BWG: body weight gain; ADFI: average daily feed intake; ADG: average daily gain; FCR: feed conversion ratio; CW: carcass weight; DP: dressing percentage; EMA: eye muscle area. BW, body weight; ADFI, average daily feed intake; ADG, average daily gain; FCR, feed conversion ratio. All data are presented as mean ± standard deviation (SD).

**Table 4:** Effect of dietary RUP ratio on meat quality of *Longissimus dorsi* in Hu sheep.

Factorial Experiment		Variables						
Protein Level (% DM)	RUP Level (% CP)	Drip Loss (%)	Shear Force (kg)	pH_45min_	pH_24h_	Eye Flesh Color at 24 h		
						L*	a*	b*
13	50	2.35 ± 0.24	7.91 ± 0.32	6.33 ± 0.11	5.67 ± 0.06	35.0 ± 1.39	19.2 ± 1.28	8.65 ± 0.83
	45	2.56 ± 0.13	8.22 ± 0.44	6.28 ± 0.14	5.64 ± 0.05	33.5 ± 1.28	20.2 ± 1.34	8.27 ± 0.43
	40	2.52 ± 0.31	7.87 ± 0.29	6.42 ± 0.23	5.71 ± 0.09	33.8 ± 1.47	19.8 ± 1.54	8.06 ± 0.38
14	50	2.43 ± 0.17	8.14 ± 0.23	6.29 ± 0.20	5.69 ± 0.07	35.1 ± 0.86	20.2 ± 1.40	7.95 ± 0.54
	45	2.68 ± 0.27	8.29 ± 0.45	6.34 ± 0.14	5.65 ± 0.04	34.1 ± 1.48	20.0 ± 1.51	8.06 ± 0.72
	40	2.52 ± 0.19	7.94 ± 0.26	6.48 ± 0.11	5.72 ± 0.07	33.7 ± 0.96	19.8 ± 1.26	8.35 ± 0.67
15	50	2.35 ± 0.24	8.35 ± 0.47	6.31 ± 0.15	5.76 ± 0.10	32.9 ± 0.87	19.7 ± 0.97	8.65 ± 0.83
	45	2.46 ± 0.13	8.21 ± 0.31	6.32 ± 0.12	5.64 ± 0.05	33.7 ± 1.30	20.3 ± 1.53	8.23 ± 0.71
	40	2.62 ± 0.31	7.88 ± 0.25	6.44 ± 0.29	5.87 ± 0.04	35.4 ± 1.27	20.2 ± 1.37	8.41 ± 0.69
16	50	2.59 ± 0.14	7.96 ± 0.33	6.39 ± 0.27	5.60 ± 0.07	35.3 ± 1.59	19.0 ± 1.12	8.17 ± 0.44
	45	2.40 ± 0.18	8.23 ± 0.42	6.37 ± 0.18	5.63 ± 0.06	33.6 ± 1.19	20.1 ± 1.04	8.46 ± 0.67
	40	2.66 ± 0.21	7.99 ± 0.31	6.45 ± 0.13	5.75 ± 0.05	33.0 ± 0.59	19.9 ± 1.34	8.33 ± 0.76
*p* value								
CP		0.268	0.218	0.288	0.492	0.378	0.58	0.625
RUP		0.328	0.178	0.258	0.369	0.310	0.519	0.347
CP*RUP		0.729	0.943	0.501	0.714	0.898	0.756	0.598

*n* = 72 (12 treatments; six sheep per treatment). All data are presented as mean ± standard deviation (SD).

**Table 5:** Effect of dietary RUP ratio on the concentration of volatile fatty acids in the rumen of fattening Hu sheep.

Factorial Experiment		Variables (mmol/100 mmol)							
Protein Level	RUP Level	Acetate	Propionate	Butyrate	Valerate	Iso-Butyrate	Iso-Valerate	Acetate/Propionate	TVFA (mmol/L)
(% DM)	(% CP)								
13	50	44.8 ± 6.14	38.5 ± 5.74	12.1 ± 3.14	2.62 ± 0.52	0.94 ± 0.41	1.09 ± 0.46	1.14 ± 0.22	71.3 ± 6.07
	45	49.6 ± 6.51	37.0 ± 3.29	8.69 ± 1.63	3.20 ± 0.21	0.47 ± 0.39	1.02 ± 0.32	1.34 ± 0.17	90.1 ± 10.80
	40	49.4 ± 5.29	37.0 ± 3.39	9.86 ± 1.95	2.08 ± 0.39	0.57 ± 0.27	1.13 ± 0.38	1.25 ± 0.13	92.5 ± 7.31
14	50	51.9 ± 6.72	32.5 ± 2.71	9.74 ± 3.76	2.56 ± 0.59	1.15 ± 0.31	1.92 ± 0.29	1.58 ± 0.25	79.4 ± 6.39
	45	50.4 ± 4.92	37.1 ± 4.16	7.3 ± 2.31	3.06 ± 0.25	0.97 ± 0.46	1.46 ± 0.25	1.23 ± 0.09	82.8 ± 7.05
	40	54.0 ± 4.36	35.5 ± 4.42	5.3 ± 2.89	3.11 ± 0.32	0.96 ± 0.33	1.01 ± 0.13	1.33 ± 0.12	78.5 ± 16.3
15	50	49.6 ± 5.65	34.8 ± 5.73	11.5 ± 1.79	1.53 ± 0.37	1.28 ± 0.42	1.37 ± 0.18	1.42 ± 0.16	73.4 ± 6.66
	45	47.0 ± 5.87	37.1 ± 2.64	11.1 ± 3.37	2.07 ± 0.46	1.14 ± 0.22	1.54 ± 0.26	1.25 ± 0.17	76.2 ± 6.28
	40	46.7 ± 6.56	38.2 ± 5.28	7.90 ± 3.82	2.21 ± 0.48	1.01 ± 0.28	1.58 ± 0.34	1.22 ± 0.29	81.9 ± 7.47
16	50	48.1 ± 6.54	36.8 ± 2.16	10.3 ± 2.63	2.30 ± 0.24	1.04 ± 0.35	1.67 ± 0.39	1.30 ± 0.31	77.6 ± 7.34
	45	46.5 ± 5.37	39.5 ± 5.37	8.70 ± 2.27	2.65 ± 0.42	0.88 ± 0.36	1.84 ± 0.42	1.17 ± 0.28	76.9 ± 6.03
	40	47.5 ± 5.82	40.8 ± 5.48	6.05 ± 1.53	2.45 ± 0.49	0.96 ± 0.47	1.85 ± 0.49	1.16 ± 0.27	81.9 ± 6.59
*p* value									
CP		0.712	0.66	0.690	0.724	0.530	0.792	0.331	0.506
RUP		0.047	0.062	0.220	0.032	0.117	<0.001	0.041	0.223
CP*RUP		0.220	0.630	0.278	0.490	0.174	0.210	0.343	0.176

*n* = 72 (12 treatments, six sheep per treatment); All data are presented as mean ± standard deviation (SD).

**Table 6:** Effect of dietary RUP ratio on volatile fatty acids of ileum in fattening Hu sheep.

Factorial Experiment		Variables (mmol/100 mmol)							
Protein Level	RUP Level	Acetate	Propionate	Butyrate	Valerate	Iso-Butyrate	Iso-Valerate	Acetate/Propionate	TVFA (mmol/L)
(% DM)	(% CP)								
13	50	57.5 ± 3.01	19.7 ± 3.11	10.5 ± 2.19	2.55 ± 0.51	5.57 ± 0.62	4.08 ± 0.51	2.92 ± 0.32	29.4 ± 3.34
	45	53.9 ± 7.02	19.7 ± 3.06	15.3 ± 2.25	1.63 ± 0.52	6.93 ± 0.61	2.61 ± 0.61	2.73 ± 0.41	30.6 ± 5.31
	40	53.6 ± 4.23	19.6 ± 2.03	12.4 ± 1.56	1.98 ± 0.60	7.98 ± 0.71	4.81 ± 0.53	2.73 ± 0.22	29.3 ± 4.41
14	50	54.7 ± 8.43	17.4 ± 1.18	13.4 ± 1.35	3.83 ± 0.64	6.72 ± 0.76	3.92 ± 0.70	3.14 ± 0.77	32.6 ± 5.58
	45	56.3 ± 7.12	19.3 ± 1.35	12.0 ± 1.74	3.20 ± 0.72	4.81 ± 0.53	4.32 ± 0.50	2.91 ± 0.35	32.2 ± 3.43
	40	53.2 ± 6.01	23.4 ± 2.21	11.5 ± 2.31	2.18 ± 0.76	5.53 ± 0.61	3.35 ± 0.52	2.29 ± 0.11	29.2 ± 3.69
15	50	54.5 ± 6.02	21.5 ± 2.35	12.3 ± 1.46	2.04 ± 0.53	4.71 ± 0.53	4.91 ± 0.64	2.20 ± 0.14	30.3 ± 5.71
	45	54.6 ± 7.91	20.8 ± 1.27	9.68 ± 2.57	1.18 ± 0.54	4.54 ± 0.52	4.20 ± 0.52	2.73 ± 0.33	35.4 ± 4.48
	40	51.9 ± 3.91	20.4 ± 3.53	7.75 ± 1.60	1.37 ± 0.67	4.18 ± 0.52	4.38 ± 0.63	2.54 ± 0.22	40.0 ± 4.58
16	50	56.2 ± 3.22	20.1 ± 3.15	13.8 ± 2.13	2.32 ± 0.78	3.53 ± 0.64	3.98 ± 0.71	2.79 ± 0.44	31.4 ± 3.74
	45	52.1 ± 5.25	22.0 ± 1.23	13.7 ± 2.09	1.72 ± 0.63	6.78 ± 0.57	3.10 ± 0.76	2.36 ± 0.49	35.4 ± 5.45
	40	58.6 ± 5.02	19.3 ± 1.42	10.8 ± 2.28	2.31 ± 0.76	4.77 ± 0.71	3.95 ± 0.58	3.03 ± 0.47	35.4 ± 5.39
*p* value									
CP		0.366	0.202	0.331	0.652	0.352	0.232	0.471	0.462
RUP		0.424	0.375	0.271	0.344	0.604	0.472	0.325	0.625
CP*RUP		0.712	0.421	0.874	0.708	0.445	0.852	0.542	0.722

*n* = 72 (12 treatments; six sheep per treatment). All data are presented as mean ± standard deviation (SD).

## Data Availability

The data supporting the conclusions of this article are included within the article. Raw data are available from the corresponding author upon reasonable request.
